# Characterization of a virulence-modifying protein of *Leptospira interrogans* identified by shotgun phage display

**DOI:** 10.3389/fmicb.2022.1051698

**Published:** 2022-11-28

**Authors:** Fabiana Lauretti-Ferreira, André Azevedo Reis Teixeira, Ricardo José Giordano, Josefa Bezerra da Silva, Patricia Antonia Estima Abreu, Angela Silva Barbosa, Milena Apetito Akamatsu, Paulo Lee Ho

**Affiliations:** ^1^Bioindustrial Division, Butantan Institute, São Paulo, Brazil; ^2^Department of Biochemistry, Institute of Chemistry, University of São Paulo, São Paulo, Brazil; ^3^Laboratory of Bacteriology, Butantan Institute, São Paulo, Brazil

**Keywords:** *Leptospira*, virulence, phage display, binding domains, protein structure prediction

## Abstract

Pathogenic species of *Leptospira* are etiologic agents of leptospirosis, an emerging zoonotic disease of worldwide extent and endemic in tropical regions. The growing number of identified leptospiral species sheds light to their genetic diversity and unique virulence mechanisms, many of them still remain unknown. Toxins and adhesins are important virulence factors in several pathogens, constituting promising antigens for the development of vaccines with cross-protection and long-lasting effect against leptospirosis. For this aim, we used the shotgun phage display technique to unravel new proteins with adhesive properties. A shotgun library was constructed using fragmented genomic DNA from *Leptospira interrogans* serovar Copenhageni strain Fiocruz L1-130 and pG8SAET phagemid vector. Selection of phages bearing new possible cell-binding antigens was performed against VERO cells, using BRASIL biopanning methodology. Analysis of selected clones revealed the hypothetical protein LIC10778, a potentially exposed virulence factor that belongs to the virulence-modifying (VM) protein family (PF07598), composed of 13 members in the leptospiral strain Fiocruz L1-130. Prediction of LIC10778 tertiary structure indicates that the protein contains a cellular-binding domain (N-terminal portion) and an unknown domain of no assigned activity (C-terminal portion). The predicted N-terminal domain shared structural similarities with the cell-binding and internalization domain of toxins like Ricin and Abrin, as well as to the Community-Acquired Respiratory Distress Syndrome (CARDS) toxin in *Mycoplasma pneumoniae*. Interestingly, recombinant portions of the N-terminal region of LIC10778 protein showed binding to laminin, collagens I and IV, vitronectin, and plasma and cell fibronectins using overlay blotting technique, especially regarding the binding site identified by phage display. These data validate our preliminary phage display biopanning and support the predicted three-dimensional models of LIC10778 protein and other members of PF07598 protein family, confirming the identification of the N-terminal cell-binding domains that are similar to ricin-like toxins. Moreover, fluorescent fused proteins also confirmed that N-terminal region of LIC10778 is capable of binding to VERO and A549 cell lines, further highlighting its virulence role during host-pathogen interaction in leptospirosis probably mediated by its C-terminal domain. Indeed, recent results in the literature confirmed this assumption by demonstrating the cytotoxicity of a closely related PF07598 member.

## Introduction

Leptospirosis is a zoonotic disease with a global widespread distribution and is currently considered an emerging infectious disease. Humans are accidental hosts and the infection by pathogenic strains of *Leptospira* generally takes place through direct contact with infected animal urine or indirectly through contaminated water (Haake and Levett, 2015). Tropical regions are highly endemic for human leptospirosis, where there is a growing uncontrolled urbanization process, leading to outbreaks often associated with poor housing and sanitation conditions, as well as extensive flooding events, contributing to the bacterial dissemination and exacerbating the risk of exposure to the population in urban and rural communities (Evangelista and Coburn, 2010; Costa et al., 2015). In Brazil, an average of 3,628 cases were confirmed between 2007 and 2020, with an average fatality rate of 9.0% per year (BRASIL, 2022).

Clinical manifestations are commonly characterized by non-specific symptoms similar to other acute febrile syndromes such as influenza, dengue, and malaria, thereby impairing its proper diagnosis. Severe leptospirosis may develop in some cases causing jaundice, acute renal failure, vascular endothelial damage, and hemorrhage, a clinical manifestation known as Weil’s disease. Leptospirosis-associated pulmonary hemorrhage syndrome is an emerging manifestation and a major cause of hemorrhagic fever in developing countries, showing a fatality rate higher than 50% (Levett, 2001; Ko et al., 2009; Marchiori et al., 2011; Dong and Chen, 2021). To prevent leptospirosis, inactivated cellular vaccines have been routinely administered to domestic and farm animals, and are used to immunize some human populations. However, these vaccines fail to promote protection against a wide range of pathogenic leptospiral serovars and were not long lasting. In an attempt to overcome the existing limitations, studies have been focused on the development of subunit vaccines, mainly consisting of surface-associated proteins conserved among serovars (Adler, 2015).

Several adhesins from a number of pathogenic bacteria have shown to generate protective antibodies in animal models, suggesting that adhesins may be potential candidates for the development of subunit vaccines, including FimH of uropathogenic *E. coli* (UPEC), the trimeric autotransporter YadA of *Yersinia enterocolitica*, outer membrane protein DsrA of *Haemophilus ducreyi*, among others (Kline et al., 2009). In addition, secreted toxins usually present cell adhesiveness through cellular receptor and are used as successful vaccine antigens, as in the case of tetanus and diphtheria vaccines (Plotkin et al., 2018). To date, leptospiral pathogenesis and its intrinsic mechanisms are not fully elucidated. Like other microbial pathogens, leptospires adhere to host cells and extracellular matrix components as a starting and necessary step for infection, allowing invasion, dissemination, and persistence in mammalian host tissues. In recent years, several *Leptospira interrogans* proteins have been described, showing binding affinities to different extracellular matrix (ECM) components *in vitro*, including leptospiral immunoglobulin-like proteins LigA and LigB, leptospiral endostatin-like protein family, LipL32, and a number of outer membrane proteins such as OmpL1 (Barbosa et al., 2006; Choy et al., 2007; Stevenson et al., 2007; Hauk et al., 2008; Castiblanco-Valencia et al., 2012; Fernandes et al., 2012; Vieira et al., 2014). Phage display has been a successful tool for identifying adhesins in many pathogenic bacteria, including *L. interrogans* proteins LIC12796, LIC11574, and LIC13411 (Lima et al., 2013; Evangelista et al., 2014; Robbins et al., 2015), besides the characterization of other binding properties of known proteins such as LipL32 and LigB (Chaemchuen et al., 2011; Ching et al., 2012). This technique was further explored in this study, leading to the identification of the conserved hypothetical protein LIC10778, as well as the characterization of its cell-binding domain.

## Materials and methods

### *Leptospira* culture and virulence maintenance

The *Leptospira* strain used in this study was the pathogenic *L. interrogans* serovar Copenhageni strain Fiocruz L1-130 (Taxonomy ID: 267671; Nascimento et al., 2004), obtained from the Faculty of Veterinary Medicine and Animal Science (University of São Paulo, SP, Brazil). Virulence and cultivation conditions were maintained as previously described (Da Silva et al., 2012). Briefly, bacteria were isolated from infected animals and cultivated in Ellinghausen–McCullough–Johnson and Harris liquid medium (Ellinghausen and Mccullough, 1965) supplemented with bovine serum albumin (BSA), under aerobic conditions at 30°C. Bacterial viability and concentration were determined by cell counting in a Petroff-Hausser chamber, under dark field microscope.

### Construction of *Leptospira interrogans* shotgun phage display libraries

Bacteriophage display libraries were constructed based on in-frame fusions to the M13 phage major coat protein encoded by gene VIII, which is present in phagemid vector pG8SAET (Jacobsson et al., 2003). The vector was digested with SnaBI restriction enzyme (New England Biolabs Inc.) for 16 h at 37°C, enabling blunt-end cloning, followed by treatment with shrimp alkaline phosphatase (SAP, Thermo Fischer Scientific) for 1 h at 37°C, in order to prevent self-ligation.

Low-passage and virulent culture of *L. interrogans* were obtained from the third and fourth passages in EMJH medium after isolation from infected hamsters. The cells were centrifuged and washed with phosphate-buffered saline (PBS) prior to genomic DNA extraction according to the DNAzol™ Reagent protocol (Thermo Fischer Scientific). Genomic DNA was then mechanically fragmented by sonication (120 s in Branson Digital Sonifier 250) and was subsequently analyzed by agarose gel electrophoresis, from which fragments containing an average of 400 base pairs (bp) were extracted using a commercially available gel extraction kit (QIAquick Gel Extraction kit, QIAGEN). Purified DNA fragments were submitted to blunting and phosphorylation reactions using T4 DNA polymerase and T4 polynucleotide kinase (Thermo Fischer Scientific), respectively, following the manufacturer’s protocols.

Size-selected *L. interrogans* DNA fragments were inserted into the previously treated pG8SAET vector using T4 DNA ligase (Thermo Fischer Scientific) in a molar ratio of 6 inserts to 1 vector. The resulting ligation mixture was electroporated into *Escherichia coli* strain TG1 cells (*supE thi-1* Δ[*lac-proAB*] Δ[*mcrB-hsdSM*]5 [r_K_^−^m_K_^−^] *F′* [*traD36 proAB* + *lacIq lacZ*Δ*M15*]) and selected on LB agar plates supplemented with 100 μg/ml ampicillin. The number of primary clones was estimated by colony counting and the proportion of transformants with *L. interrogans* DNA insert was assessed by performing colony PCR and DNA sequencing using pG8SAET-specific oligonucleotides (Supplementary Table S1) on randomly picked colonies.

### Selection of bacteriophage clones (biopanning) in VERO cells

VERO epithelial cells isolated from the kidney of *Cercopithecus aethiops* monkeys (Simizu et al., 1967) were chosen for biopanning of selected phage library and cell-binding assays, as they are derived from one of the main organs affected by pathogenic *Leptospira* infection. Cells were cultured in Dulbecco’s Modified Eagle Medium (DMEM high glucose, Gibco™), supplemented with antibiotics (Penicillin/Streptomycin solution, Gibco™; 100 mg/1 l DMEM) and 10% fetal bovine serum (FBS, Gibco™), at 37°C and humidified 5% CO_2_.

After evaluation of the obtained shotgun phage display libraries, the best library was chosen for selection of ligand phages in VERO cells, following the biopanning and rapid analysis of selective interactive ligands methodology (termed BRASIL). This approach relies on separating cell-bound phages from phages in suspension through an organic and hydrophobic phase, which helps to remove water-soluble substances surrounding the cell surface. Cell-bound phages cross the lower organic phase during centrifugation and can be recovered in the pellet, while unbound phages remain in the upper aqueous phase, avoiding its undesirable recovery (Giordano et al., 2001).

Biopanning using BRASIL technique as performed with some modifications. Briefly, a shotgun phage display library was electroporated into *E. coli* TG1 cells and the resulting culture was infected with M13KO7 helper phage for assembly, amplification, and production of the recombinant phages (Barbas et al., 2001). Concomitantly, confluent VERO cells were washed three times with PBS plus 10 mM EDTA and harvested. After centrifugation at 150 x *g* for 5 min at 4°C, cells were suspended in DMEM plus 1% BSA and cell concentration was adjusted to 10^7^ cells/ml. Aliquots of 100 μl containing 10^6^ cells were incubated with 100 μl of recombinant phages for 3 h at 4°C. The resulting mixture was gently added to 200 μl of an organic phase (9:1 volume proportion of dibutyl phthalate:cyclohexane) and centrifuged (10,000 x *g* for 10 min at 4°C). The bottom of the tube containing cell pellet was cut and transferred to a new tube, in which the cell-bound phages were suspended in 500 μl of previously prepared *E. coli* TG1 culture in TB medium (OD_600nm_ 1–1.2). The mixture was incubated for 30 min at room temperature, and subsequently, the infected culture was amplified to recover phages for a new round of selection. In total, three rounds of selection were carried out and the enrichment of binding phages was assessed by phage titration after each round.

### Analysis of selected phage clones

Proteins of interest for this study are putative adhesins or molecules presenting cell-binding activity that might display a transmembrane region or a signal peptide in its sequence in order to be exposed on the bacterial surface or secreted to its surroundings to target cellular receptors or ECM components. Colonies infected with selected phages were harvested after each round for phage clone analysis by PCR and DNA sequencing. The resulting data were further analyzed by DNA and protein sequence alignment tools (see Supplementary Table S2) to find proteins encoded by leptospiral genomic DNA in frame with the phage protein encoded by gene VIII, allowing its presentation on the phage surface. Prediction programs (described in Supplementary Table S2) were then applied to analyze the selected leptospiral proteins for the presence of signal peptides, transmembrane regions, possible subcellular localization, and domains.

### Expression of *lic10778* gene by RT-qPCR during infection

Liver samples from three C3H/HeJ mice infected intraperitoneally with 3 × 10^7^ cells of virulent *L. interrogans* serovar Copenhageni strain Fiocruz L1-130 were removed after 3 days of infection, immediately frozen in liquid nitrogen, and stored at −80°C until the extraction of total RNA, using Trizol reagent (Invitrogen). Subsequently, extracted RNA was treated with DNase (Thermo Fischer Scientific) at 37°C for 30 min, followed by reverse transcription reaction using SuperScript III (Invitrogen) to obtain cDNAs. As a control, RNA was purified from *L. interrogans* cultured for 5 days in EMJH medium at 30°C and transcribed to cDNA according to the same protocols described above. qPCR reactions were performed in triplicate on the Applied Biosystems 7,300 Real-Time PCR System (Applied Biosystems), using SYBR® Green Master Mix reagent (Thermo Fischer Scientific), cDNA from liver or control samples and specific oligonucleotides (Supplementary Table S1). After 40 PCR cycles and data extraction, expression levels of the selected genes were analyzed from the cycle threshold (*C*_T_) values, and compared to the *C*_T_ values obtained with the expression of ribosomal 16S gene for normalization. Relative gene expression to the control condition (culture in EMJH medium at 30°C) was measured using the delta–delta method expressed in 2^–ΔΔCT^ equation (Livak and Schmittgen, 2001; Pfaffl, 2001).

### Statistical analysis

Gene expression data from qPCR were further analyzed by One-way ANOVA followed by Tukey’s multiple comparison test using GraphPad Prism Software, version 5.03 for Windows.

### Three-dimension structural analysis and modeling of LIC10778 protein

LIC10778 amino acid sequence was used for prediction of the protein three-dimensional structure (Supplementary Table S3), in order to better guide the design of new recombinant proteins for further functional analysis, or to compare the models found with the results already obtained.

The Phyre2 server uses a combination of modeling with multiple protein templates already known interspersed with regions simulated in an *ab initio* way, while SWISS-Model and HHpred programs use homology detection to predict the structure of proteins of interest. To compare predicted and deposited protein structures, the root mean square deviation (RMSD) of the atomic positions was calculated after structural alignment using the MatchMaker tool of UCSF Chimera software. A more recent methodology for predicting protein structures based on deep learning was also used to analyze LIC10778 protein: RoseTTAFold (Robetta server) allows the generation of highly accurate models from a three-way neural network capable of simultaneously processing the amino acid sequence, distance between residues and information about their coordinate properties (Baek et al., 2021). All generated models were subsequently modified in PyMol software to assign specific colors and delimit each portion or predicted domains of the LIC10778 protein.

### Cloning, expression, and purification of LIC10778 recombinant proteins

Gene sequences of LIC10778 (LIC10778F, LIC10778_LecB, LIC10778_Phg, LIC10778_D1, LIC10778_Cards, LIC10778_Ct and EGFP-LIC10778_Cards), LIC12339 (LIC12339F), and LIC10870 (LIC10870F) were amplified by high-fidelity PCR using *L. interrogans* sv. Copenhageni str. Fiocruz F1-130 genomic DNA as template, specific oligonucleotide pairs (Supplementary Table S1), and Phusion™ High-Fidelity DNA Polymerase kit (Thermo Fischer Scientific). Resulting PCR products were subjected to adenylation and cloning in the pGEM®-T Easy Vector System (Promega) for subsequent cleavage with appropriate restriction enzymes to generate non-compatible cohesive overhangs for direct subcloning into bacterial expression vectors, previously digested with the same enzymes. Expression vectors used in this study were pAE (Ramos et al., 2004), and pAZ (Butera et al., 2005), which was used for the expression of recombinant fluorescent proteins (Enhanced Green Fluorescent Protein [EGFP] and EGFP-LIC10778_Cards). Recombinant plasmids were amplified in *E. coli* DH10B bacteria selected in LB medium supplemented with 100 μg/ml ampicillin and isolated using a commercially available kit (QIAprep® Spin Miniprep Kit, QIAGEN). After sequence analysis by DNA sequencing, plasmids with confirmed inserts were used to transform competent *E. coli* BL21 Star (DE3) pLysS, *E. coli* BL21-SI, or *E. coli* ArcticExpress (DE3) cells, and the production of 6xHis-Tag fusions in 1-liter *E. coli* cultures was performed according to each strain requirements (see Table 1). Bacterial cells were harvested by centrifugation and lysates were prepared by pressing the cells at 1,000–1,300 bar (GEA Lab Homogenizer PandaPLUS 2000). Recombinant proteins were purified by immobilized-metal affinity chromatography (IMAC) using HisTrap HP columns (GE Healthcare Life Sciences).

**Table 1:** Bacterial strains used and corresponding culture conditions for expression vector amplification and production of recombinant proteins.

*Escherichia coli* strain	Antibiotics	Conditions for protein expression induction
BL21 Star (DE3) pLysS	Chloramphenicol (30 μg/ml)	0.5–1 mM IPTG
	Expression vector resistance (100 μg/ml ampicillin)	30°C, 3–4 h
BL21-SI	Expression vector resistance (100 μg/ml ampicillin)	200–300 mM NaCl
		30°C, 3–4 h
ArcticExpress (DE3)	Gentamycin (20 μg/ml)	0,05–0,5 mM IPTG
	Streptomycin (75 μg/ml)	10–12°C, 24 h
	Expression vector resistance (100 μg/ml ampicillin)	

Recombinant proteins LIC10778F, LIC10870F, and LIC12339F were produced in *E. coli* BL21 Star (DE3) pLysS bacteria, while the recombinant proteins LIC10778_Cards, LIC10778_D1, LIC10778_LecB, LIC10778_Phg, and LIC10778_Ct were produced in *E. coli* BL21-SI bacteria. All proteins were obtained from inclusion bodies, requiring dissolution of the proteins in 8 M urea or 6 M guanidine hydrochloride solutions, following purification on HisTrap HP columns. EGFP and EGFP-LIC10778_Cards were produced in *E. coli* ArcticExpress (DE3) in soluble form and were also purified on HisTrap HP columns. Recombinant LigA and LIC10301 were obtained as described in the literature (Barbosa et al., 2010; Castiblanco-Valencia et al., 2012). Recombinant proteins were visualized by SDS-PAGE gel electrophoresis after Coomassie blue staining.

### Protein interaction with ECM components by far Western (overlay) blotting

Interaction of recombinant proteins (LIC10778 fragments and other proteins of the PF07598 family) with ECM components was evaluated by the Far Western (Overlay) Blotting technique (Wu et al., 2007; Hall, 2015). First, recombinant proteins LIC12339F, LIC10870F, LIC10778F, LIC10778_LecB, LIC10778_Phg, LIC10778_D1, LIC10778_Cards, and LIC10778_Ct purified by IMAC in 8 M urea were diluted in sample buffer for SDS-PAGE and applied to gels prepared with two concentrations of polyacrylamide (9 and 16%) for separation under voltage of 80–130 V. Recombinant proteins LigA and LIC10301 were also applied to the gels as positive and negative adhesin controls, respectively. After electrophoretic migrations, proteins were transferred to nitrocellulose membranes (Hybond ECL, GE Healthcare Life Sciences) at 10 V. Protein transfer was evaluated after membrane incubation with Ponceau S dye. Subsequently, membranes were incubated with blocking solution (PBS-T with 5% BSA) at 4°C for approximately 16 h. After PBS-T wash, membranes were incubated in solutions containing one of the following components of the ECM or plasma, diluted to 10 μg/ml in PBS-T: collagen type I from rat tail (Corning), laminin and collagen type IV from Engelbreth–Holm–Swarm murine sarcoma basement membrane, human fibroblast cell fibronectin, human plasma fibronectin, 30 kDa and 45 kDa proteolytic fragments of human plasma fibronectin and vitronectin (Sigma-Aldrich). After incubation, membranes were washed 4 times with PBS-T and incubated with specific primary and polyclonal antibodies against the ECM or plasma proteins (Sigma-Aldrich), diluted at 1:2,500 in PBS-T for 1 h at RT. Membranes were washed again, followed by incubation with peroxidase-conjugated anti-rabbit IgG secondary antibody (Sigma-Aldrich) diluted 1:7,000 in PBS-T for 1 h at room temperature. Finally, interactions were evaluated using detection kit solutions A and B (Amersham® ECL Prime WB Detection Reagent, GE Healthcare Life Sciences), following luminescence detection and documentation using a photodocumenter (Kodak Gel Logic 2,200 Imaging System, Carestream).

### Cell-binding assay with fluorescent proteins

Binding of fluorescent recombinant EGFP-LIC10778_Cards protein to cells was performed in VERO and pulmonary A549 cells. Confluent cultures were added to 24-well microplates (Corning®) at 2 × 10^5^ cells/well in complete DMEM (supplemented with antibiotics and 10% FBS) and incubated at 37°C, 5% CO_2_. After 24 h of incubation, cells were washed once with PBS and incubated in serum-free DMEM for 5 h. Duplicate wells of adherent cells were incubated with 10–15 μg/well of recombinant EGFP-LIC10778_Cards samples or EGFP alone as control diluted in serum-free DMEM supplemented with 1% BSA for 2 h at 37°C under 5% CO_2_. Unbound proteins were washed with PBS buffer containing calcium (132.5 mg/L) and magnesium (100 mg/L) and cells were fixed with 4% paraformaldehyde in PBS for 30 min at RT. After a washing step with the same PBS buffer, wells were further incubated with DAPI dye for nuclear staining. After final wash, cells were conserved in 90% glycerin solution for fluorescence evaluation, using EVOS M5 fluorescence microscope.

## Results

### Evaluation of bacteriophage libraries and selection of a LIC10778 fragment

Bacteriophage libraries were constructed with the genomic DNA from *L. interrogans* in order to obtain a suitable number of primary clones, i.e., those containing unique inserts, for shotgun phage display. A good quality library has to be large enough to cover the entire bacterial genome and to overcome the relatively low proportion of clones with correct inserted fragments for proper display of fused proteins (Jacobsson et al., 2003; Mullen et al., 2006). For this reason, we established an ideal minimum number of clones around 2.07×10^7^ clones, considering the genome extension of *L. interrogans* strain used (4.6 Mb), the probability of correct fragment insertion (1/18), the medium size of genomic DNA fragments (400 pb), and a sufficient coverage of the genome (100 times). Constructed libraries were evaluated by the total number of primary and valid clones, that is, those that had the insertion of a fragment of *L. interrogans* genome. The chosen library showed 2.26 × 10^7^ valid clones, thus surpassing the expected threshold number of clones in terms of genome representation.

**Figure 1:**
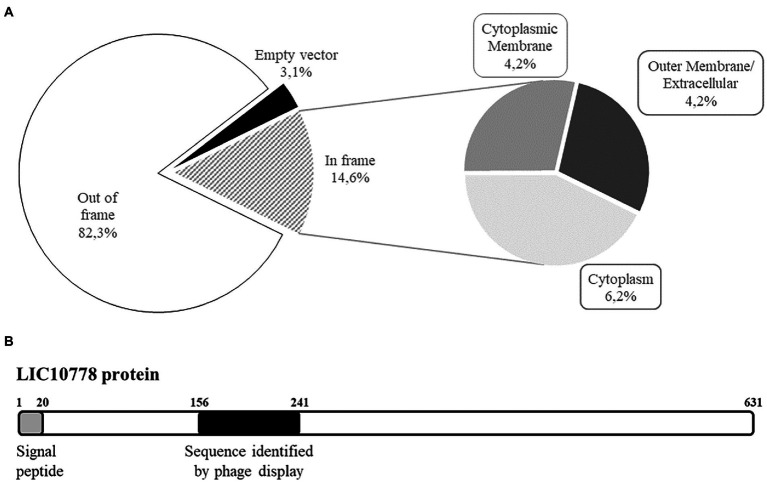
Shotgun phage display led to the identification of LIC10778 protein. **(A)** Classification of shotgun library phages bound to kidney epithelial cells (VERO) along three rounds of selection using BRASIL methodology. Samples were evaluated by alignment (BLASTx) and by determination of the six possible reading frames (EMBOSS Transeq). Subsequently, clones were classified based on the presence of *Leptospira interrogans* sequences displayed “In frame” or “Out of frame” with pVIII bacteriophage protein. Clones that lacked *L. interrogans* sequences were classified as “Empty vector.” In-frame clones were further classified according to their predicted subcellular location in leptospires: “Cytoplasmic membrane,” “Outer membrane/Extracellular,” or “Cytoplasm.” **(B)** Schematic representation of LIC10778 protein, showing the predicted signal peptide and the sequence identified by phage display, both located at the N-terminal region of the protein.

Leptospiral proteins that potentially interact with VERO kidney epithelial cells were enriched along three rounds of selection by BRASIL technique, a single-step centrifugation approach that eliminates the need to perform repeated washes. Samples containing recombinant phages bound to cells were harvested for analysis by sequencing and subsequently classified according to the insert reading frame in relation to the gene encoding pVIII phage protein present in the phagemid pG8SAET. From the 96 individual cell-bound phages sequenced, most clones had inserts out of frame and a few clones were empty vectors (Figure 1A). The remarkable selection of clones out of frame was also observed in previous studies and it can be explained by the formation of artificial open reading frames that act as mimotopes or by ribosomal slippage that can correct the frameshift during translation (Cárcamo et al., 1998; Fehrsen and du Plessis, 1999). On the other hand, 14 clones (14.6%) were correctly fused to pVIII (Figure 1A) and *in silico* analysis by sequence alignment and structure prediction programs revealed the selection of sequences from cytoplasmic proteins (6 out of 96, 6.2%), cytoplasmic membrane proteins (4 out of 96, 4.2%), and outer membrane/extracellular proteins (4 out of 96, 4.2%). Among the latter group, we identified a single clone with the peptide related to the conserved hypothetical protein LIC10778 (Figure 1B), selected among enriched phage clones from the third round of selection in VERO cells.

### *In silico* analysis of LIC10778 and PF07598 protein family

LIC10778 protein contains 631 amino acid residues and a predicted N-terminal signal peptide typical of secreted proteins by the Sec/SPI pathway revealed by prediction programs, being a protein probably secreted to the extracellular environment. The sequence identified by shotgun phage display corresponds to the peptide sequence between amino acid residues 156 and 241, located at the N-terminal region of the protein (Figure 1B). The most striking feature of LIC10778 protein is that it belongs to a protein family in leptospires with similar sequences, clustered by the domain of unknown function DUF1561 (Pfam: PF07598), also known as the Virulence Modifying (VM) protein family (Fouts et al., 2016).

According to the InterPro tool, the PF07598 motif is present in 818 proteins only in the Eubacteria kingdom: less than 0.4% of these proteins are present in the Actinobacteria and Firmicutes clades, while 15.4% were present in the Proteobacteria clade, including units in the genera *Helicobacter*, *Campylobacter*, *Bartonella*, *Piscirickettsia* and in the family *Vibrionaceae*. More than 84% of the PF07598 domain is present in pathogenic species of the genus *Leptospira*, especially those more virulent and capable of causing the most severe forms of leptospirosis in humans (Lehmann et al., 2013; Fouts et al., 2016; Picardeau, 2017). Just like LIC10778, most of PF07598 protein family members have a secretory signal peptide and/or transmembrane region, and all proteins are predicted to be secreted by leptospires, being localized extracellularly or attached to the outer membrane (Supplementary Table S4). Another remarkable feature shared among these proteins is the presence of 12 Cys residues at invariable positions (Supplementary Figure S1), suggesting a conserved pattern of disulfide bond formation that may contribute to an extracellular function (Kadokura et al., 2003; Landeta et al., 2018). Interestingly, the LIC12985 protein is an unusual member of the PF07598 protein family found in *L. interrogans* strain Fiocruz strain L1-130, which corresponds only to the C-terminal region of the other members of this protein family.

### High expression of *lic10778* gene in susceptible infected animals

**Figure 2:**
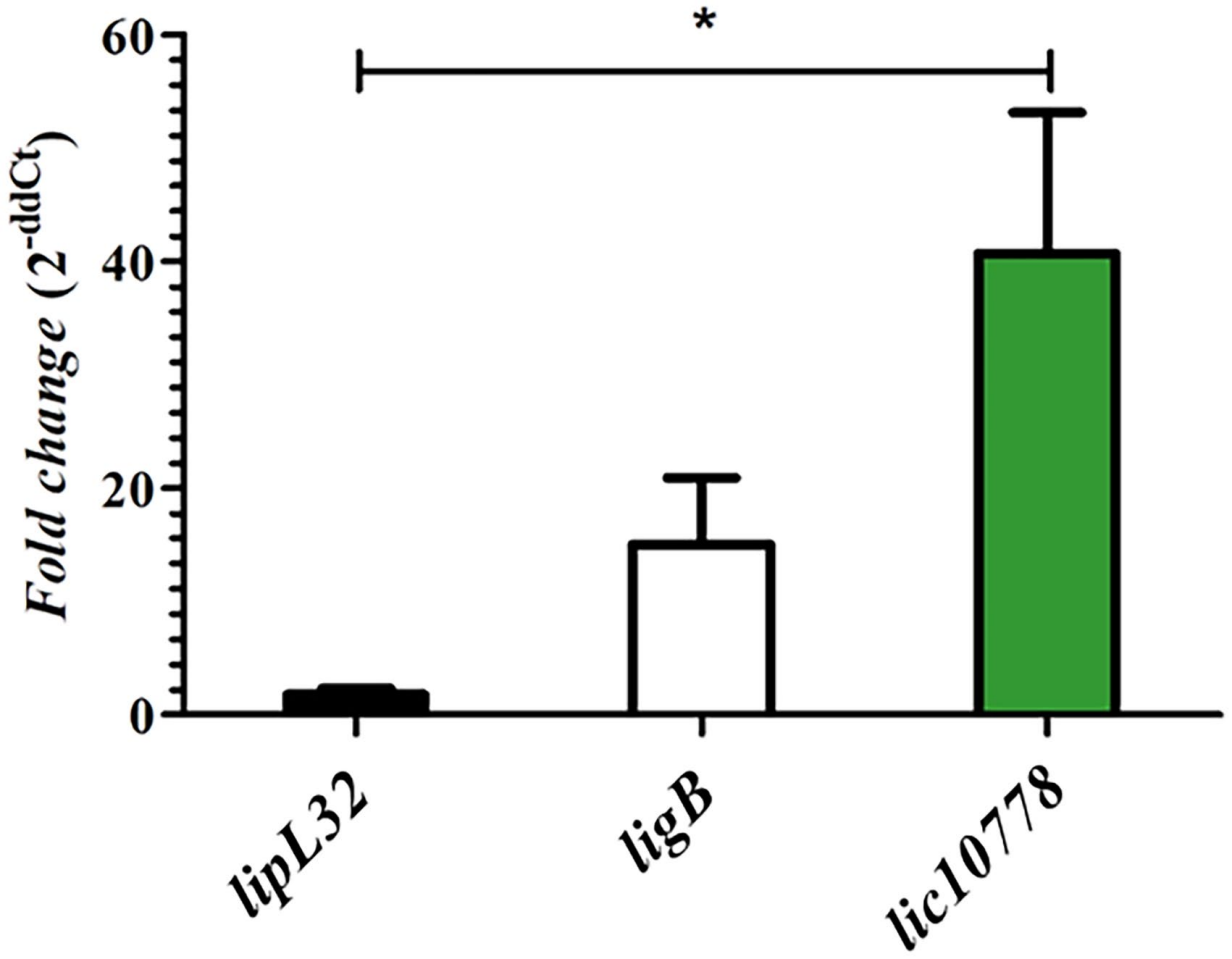
*lic10778* gene is highly expressed in susceptible infected animals. RT-qPCR analysis of the *lic10778* gene expression, *lipL32* and *ligB* genes as controls, presented in fold change measured by the delta–delta method (2^-ddCt^). cDNA samples were obtained from liver of C3H/HeJ susceptible mice after 3 days of infection by *L. interrogans* serovar Copenhageni strain Fiocruz L1-130. Samples were analyzed in triplicate for each oligonucleotide pair, and data were normalized by 16S ribosomal gene expression. Relative expression was compared to the expression of the same genes in leptospires cultured in EMJH medium at 30°C. Statistical analysis by One-way ANOVA, with *p <* 0.05 (*).

The presence of PF07598 protein family in pathogenic species of leptospires and the relationship of their members to host interaction and virulence have been demonstrated in previous publications (Matsunaga et al., 2007; Lehmann et al., 2013; Marcsisin et al., 2013; Chou et al., 2014; Putz et al., 2021). In particular, PF07598 protein family was initially explored in *L. interrogans* serovar Lai. Lehmann et al. identified non-synonymous single nucleotide variants (nsSNVs) in two genes encoding PF07598 proteins, including the orthologous gene of *lic10778* (*la_3388*), when comparing wild-type and culture-attenuated leptospiral genomes. The same study revealed an up-regulated expression of all family members to varying degrees in blood, liver, and kidney from hamsters acutely infected with virulent leptospires. The orthologous gene of *lic10778* was one of the most highly up-regulated genes (Lehmann et al., 2013, 2014). Based on the literature data, a qPCR reaction was performed with specific oligonucleotides for *lic10778* gene and cDNA obtained from RNA extracted from the liver of susceptible mice C3H/HeJ after 3 days of the infection with virulent *L. interrogans* serovar Copenhageni strain Fiocruz L1-130. The increase in *lic10778* gene expression was confirmed, resulting in a mean 40-fold increase compared to the basal expression level in EMJH culture. This increase was significant in relation to the expression of *lipL32*, while the difference between the expression of ligB and lic10778 was not significant (Figure 2).

### Predicted N-terminal domain of LIC10778 is structurally similar to cell-binding and internalization domains of CARDS and ricin-like toxins

Initial attempts to predict the LIC10778 tridimensional structure were performed using Phyre2, Swiss-Model, HHpred programs. All the programs suggested structural similarity with the carbohydrate-binding domains of several proteins, which have similar folding to the ricin B lectin domain. The N-terminal portion of the LIC10778 protein (41–155 residues) was modeled with more than 90% confidence, with the lectin B domain of abrin toxin (PDB 1abr), which is also confirmed by the RMSD of 0.864 Å after aligning the two structures (Supplementary Figure S2). Detailed analysis of this sequence highlights shared features, such as the position of four cysteine residues and the QxW or similar repeats, which are hallmarks of ricin B-like lectin domain (Rutenber et al., 1987; Hazes, 1996). Interestingly, all the PF07598 proteins in the Fiocruz L1-130 strain (except for LIC12985) shows a remarkable similarity in this N-terminal region (Supplementary Figure S2).

**Figure 3:**
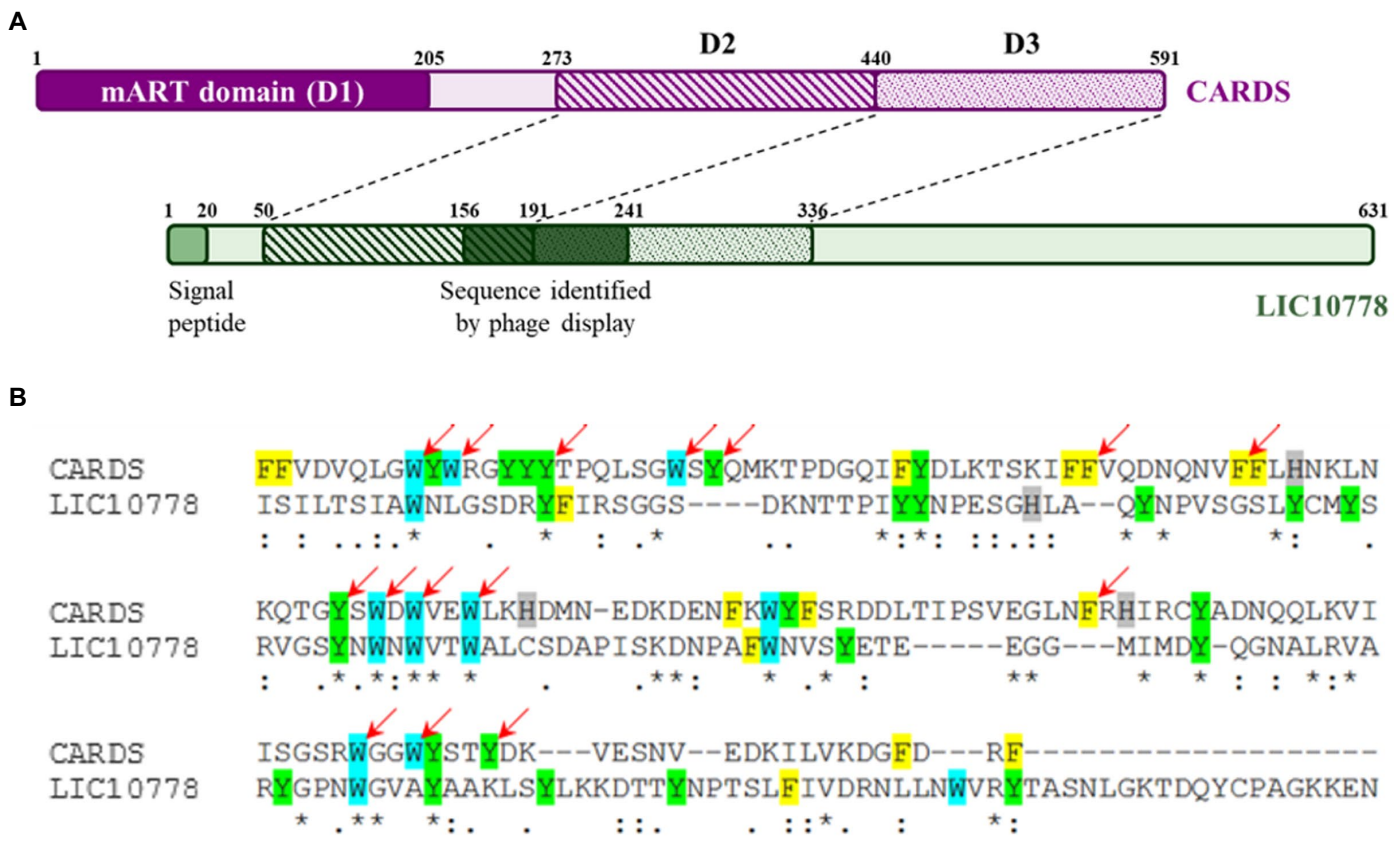
LIC10778 alignment with CARDS toxin. **(A)** Schematic representation of CARDS toxin from *Mycoplasma pneumoniae* (in purple) and its D2 and D3 domains aligned with LIC10778 protein (in green). **(B)** Alignment detail of CARDS toxin D3 domain with LIC10778 sequence (191–336 aa), where the aromatic residues are highlighted in yellow (phenylalanine), cyan (tryptophan), green (tyrosine) or grey (histidine). Red arrows indicate residues that form the “aromatic patch,” essential for CARDS toxin-binding and internalization function in cells.

In addition to the similarity with the carbohydrate-binding domains, the SWISS-Model and HHpred programs also showed structural alignment of approximately 40% of the LIC10778 protein with the PDB 4tlw and 4tlv models, corresponding to the CARDS toxin of *Mycoplasma pneumoniae*, occurring between the N-terminal portion of LIC10778 and the full extent of D2 and D3 domains of CARDS toxin (Figure 3A). D2 and D3 domains of CARDS toxin are structured in three β sheets, similar to ricin toxin domains (Rutenber and Robertus, 1991). Together with toxin binding and internalization into cells, these domains are also responsible for toxin-mediated vacuolization activity. Literature data show that D3 domain is especially necessary for full activity of the toxin, possibly mediated by an “aromatic patch” formed within the domain: of the 152 amino acid residues, 35 are aromatic (23%) and 15 of them are part of the D3 domain “aromatic patch” (Kannan et al., 2014; Becker et al., 2015). LIC10778 sequence aligned with CARDS toxin D3 domain has 25 aromatic residues (17% of the 146 total amino acids), of which 14 of them align with the aromatic amino acids of the CARDS toxin and 7 of them coincide with the amino acids belonging to the “aromatic patch” (Figure 3B).

Indeed, using the more recent and accurate tool of deep learning RoseTTAFold (Robetta server), the structural model for LIC10778 protein was generated, confirming the observed features from the previous prediction programs. In the same way, models for all other 12 members of the PF07598 protein family present in *L. interrogans* serovar Copenhageni strain Fiocruz L1-130 were also generated (Supplementary Figure S3). Despite some small structural differences, it is possible to observe the great similarity of the 13 models to each other. All models show a tendency to separate the N- and C-terminal domains and present similarity with the cellular-binding and internalization domains of toxins such as CARDS, abrin, and ricin.

### Design, production, and purification of LIC10778 recombinant proteins

**Figure 4:**
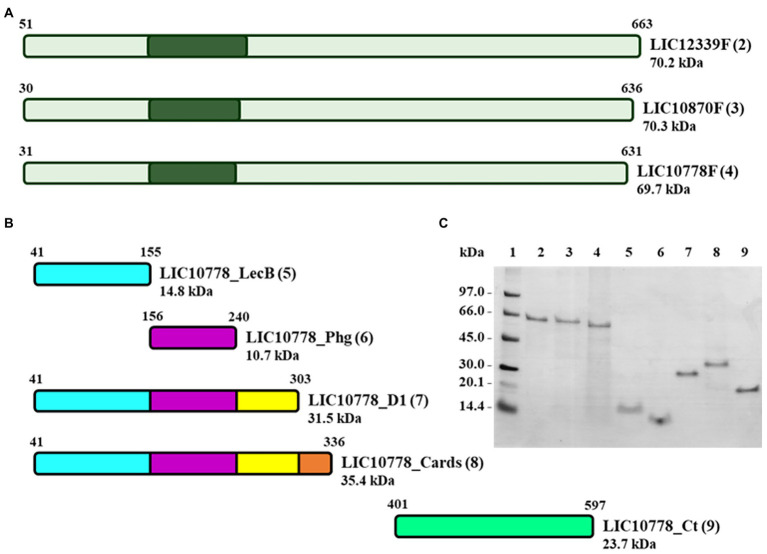
Recombinant proteins of PF07598 protein family members LIC10778, LIC10870, and LIC12339. **(A)** Scheme of the full-length recombinant proteins corresponding to LIC12339, LIC10870, and LIC10778 protein sequences (LIC12339F, LIC10870F, and LIC10778F, respectively). Dark green areas represent the location of the sequence identified by phage display. **(B)** Scheme of five recombinant proteins of LIC10778, designed from the three-dimensional models generated by prediction programs and identified by the following suffixes: _LecB, referring to the lectin B domain; _Phg, referring to the sequence identified by phage display; _D1, referring to the N-terminal domain delimited by the Phyre2 server; _Cards, referring to the sequence aligned to the CARDS toxin of *M. pneumoniae*; _Ct, referring to the C-terminal region of the protein. **(C)** SDS-PAGE with the recombinant proteins purified by IMAC in 8 M urea or 6 M guanidine hydrochloride solutions. Proteins were applied in lanes 2 to 9, the same numbers as in each protein scheme shown in panels **A** and **B** (numbers in parentheses). Lane 1: molecular mass marker. SDS-PAGE with 16% plus 9% polyacrylamide gel followed by Coomassie Blue staining.

Recombinant proteins were designed considering the full length of LIC10778, as well as for LIC10870 and LIC12339 proteins (Figure 4A), which represent proteins of greater and lesser identity with the consensus sequence resulting from the multiple sequence alignment of the 13 PF07598 members in the Fiocruz L1-130 strain (Ferreira, 2015; Supplementary Figure S1). In addition to these constructions, the design of new recombinant fragments of LIC10778 protein was defined from its three-dimensional model generated with the aid of prediction programs, in order to confirm possible domains and their associated functions: LIC10778_Cards, corresponding to the sequence aligned to D2 and D3 domains of CARDS toxin; LIC10778_D1, corresponding to the possible N-terminal domain of the LIC10778 protein (Phyre2 server prediction); LIC10778_LecB, corresponding to the ricin B lectin domain; LIC10778_Phg, corresponding to the sequence presented by the selected phage after cell selection (phage display and BRASIL techniques); LIC10778_Ct, corresponding to a C-terminal sequence of the LIC10778 protein (Figure 4B).

Production and purification of the recombinant proteins were evaluated by SDS-PAGE, in which each recombinant protein presented a molecular mass close to that expected by the predictions and showed that its purification was successful (Figure 4C).

### N-terminal portion of LIC10778 interacts with ECM proteins

ECM components are closely associated with cells in different tissues and organs, providing structural support, contributing to basic cellular processes such as proliferation, differentiation, and migration, in addition to representing binding targets of various pathogens during the infectious process (Sorokin, 2010). In order to evaluate the interaction between recombinant proteins of PF07598 protein family members LIC10778, LIC10870, and LIC12339 with ECM and plasma proteins, Far Western Blotting assays (also known as Overlay) were performed.

**Figure 5:**
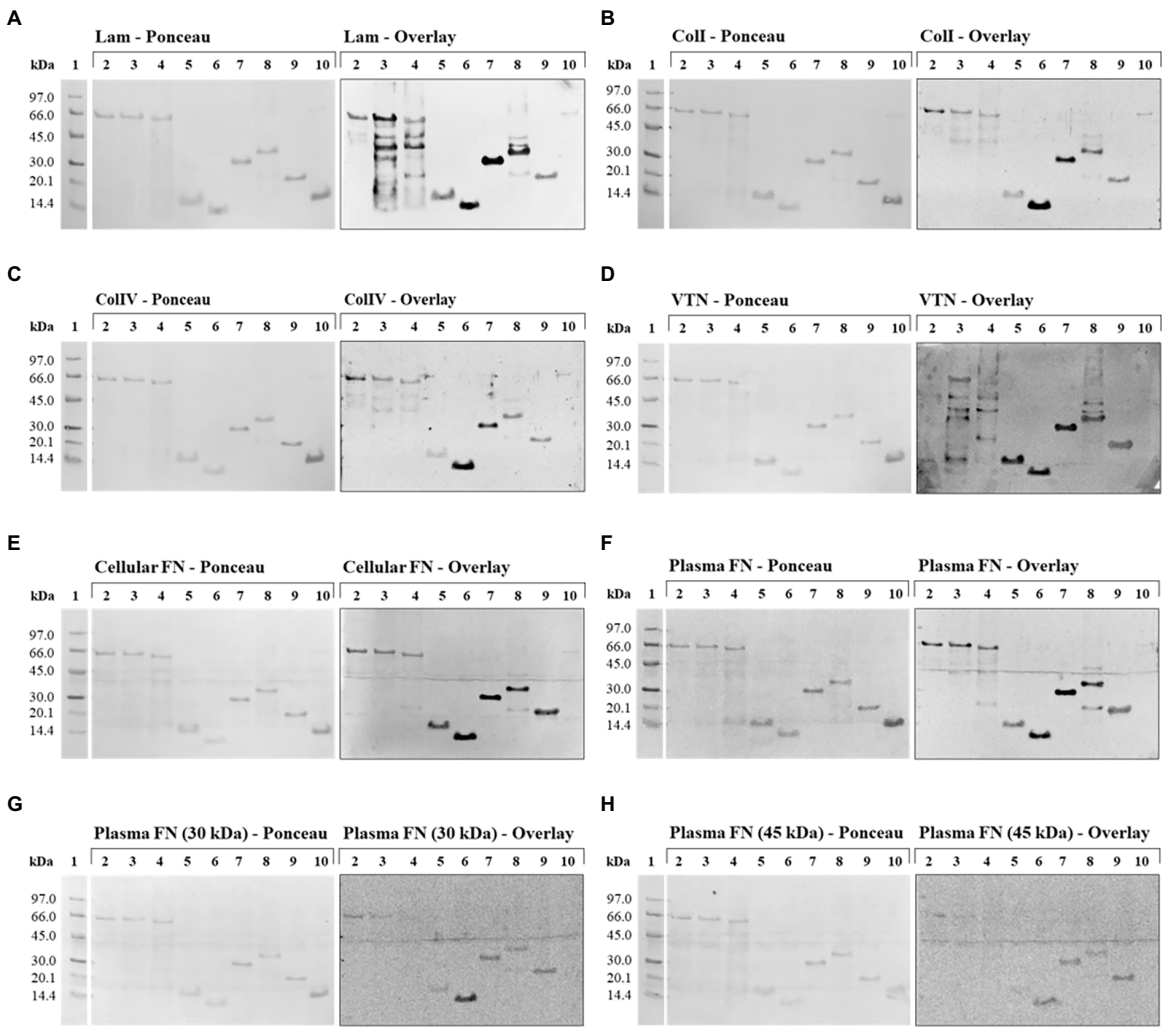
Far Western Blotting (Overlay) assay with LIC10778, LIC12339, and LIC10870 recombinant proteins, and their interaction with ECM proteins. On the *left* are the nitrocellulose membranes stained with Ponceau S, after SDS-PAGE fractionation and transfer, with the recombinant proteins in the following order: LIC12339F (lane 2), LIC10870F (lane 3), LIC10778F (lane 4), LIC10778_LecB (lane 5), LIC10778_Phg (lane 6), LIC10778_D1 (lane 7), LIC10778_Cards (lane 8), and LIC10778_Ct (lane 9). In lane 10, LigA (63 kDa) and LIC10301 (13 kDa) were added as positive and negative controls, respectively. On the *right* are the chemiluminescent signals observed after incubation of the corresponding nitrocellulose membranes with 10 μg/ml of the proteins: laminin (Lam, **A)**, collagen I (ColI, **B)**, collagen IV (ColIV, **C)**, vitronectin (VTN, **D)**, cellular fibronectin (cellular FN, **E)**, plasma fibronectin (plasma FN, **F)** or 30 kDa **(G)** and 45 kDa **(H)** proteolytic fragments of plasma fibronectin, followed by specific antibodies to each of these proteins. Lane 1: molecular mass marker. Results were obtained from two independent assays.

With the exception of the negative control LIC10301, all recombinant proteins showed interactions at different levels with one or more ECM proteins used (Figure 5), but it is possible to observe the detection signals of all recombinant proteins referring to the N-terminal region of LIC10778 protein, especially the sequence selected by phage display. In almost all membranes evaluated, the LIC10778_Phg protein is highlighted, indicating its interaction with ECM proteins, except with the 45 kDa proteolytic fragment of plasma fibronectin. LIC10778_D1 protein also showed a remarkable interaction with laminin, collagen I, vitronectin, and with cellular and plasma fibronectins. LIC10778_Cards protein interacted slightly less with laminin and cellular and plasma fibronectins, while LIC10778_LecB fragment interacted similarly with laminin, vitronectin, and cellular fibronectin. As for the recombinant proteins corresponding to full-length sequences of PF07598 protein family members, their interaction with ECM proteins was more discreet compared to the LIC10778 fragments, with the exception of the interaction of LIC10870F and LIC10778F with laminin. These results suggest that the N-terminal region (including the identified phage-displayed sequence) is the main portion of LIC10778 that interacts with the host, being the fragment identified by phage display presenting the highest interaction with most of the ECM proteins analyzed.

### N-terminal portion of LIC10778 binds to VERO and A549 cells

**Figure 6:**
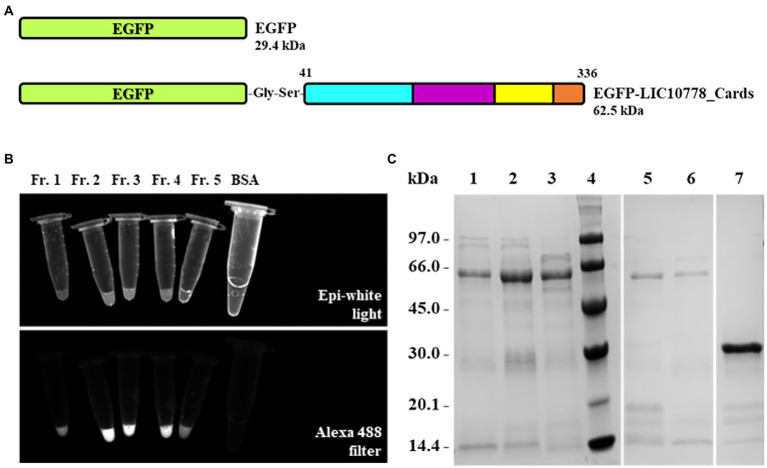
Expression and purification of EGFP-LIC10778_Cards protein. **(A)** Protein scheme of the recombinant proteins cloned into the pAZ expression vector corresponding to the sequences of the EGFP alone and the EGFP-LIC10778_Cards proteins. The portion of LIC10778 protein in this fusion has the same amino acid sequence as shown in Figure 4B. **(B)** Fluorescence evaluation of EGFP-LIC10778_Cards protein purified samples under exposure to Alexa 488 filter in ChemiDoc photodocumenter (Bio-Rad), in comparison to epi-white light exposure. Fractions 1 to 5 (Fr. 1–5) represent different elution fractions of protein purification on HisTrap column. A sample containing BSA was also evaluated as a negative control. **(C)** SDS-PAGE with the fractions of the recombinant proteins purified by IMAC. Samples of EGFP-LIC10778_Cards fused protein denominated as fractions 1 to 5 are represented in lanes 1, 2, 3, 5, and 6, respectively, recombinant EGFP were added in lane 7. Lane 4: molecular mass marker. SDS-PAGE with 12% polyacrylamide gel stained with Coomassie Blue.

The LIC10778_Cards recombinant protein that presents the entire N-terminal fragment of LIC10778 had its nucleotide sequence cloned in fusion with EGFP gene for the production of EGFP-LIC10778_Cards protein (Figure 6A). In order to confirm the interaction of this portion of the LIC10778 to mammalian cells, EGFP alone and EGFP-LIC10778_Cards proteins were produced in *E. coli* cells. After purification and dialysis, samples of recombinant EGFP protein were clearly greenish, indicating successful expression. Samples of EGFP-LIC10778_Cards protein did not show a green color by visual inspection, but most of them showed fluorescence under exposure to the Alexa 488 filter in photodocumenter (Figure 6B). All soluble recombinant proteins were further evaluated by SDS-PAGE (Figure 6C).

Next, EGFP-LIC10778_Cards samples were incubated with VERO and A549 cells in culture previously deprived of fetal bovine serum. VERO kidney cells were chosen because of their previous use in the selection of recombinant phages in phage display assays, in addition to be derived from an important targeted organ of leptospires during infection. A549 cells are human lung epithelial cells, which have some CARDS toxin-binding receptors (Kannan et al., 2014; Somarajan et al., 2014), which are also possible binding targets of pathogenic leptospires through LIC10778 and related protein members.

**Figure 7:**
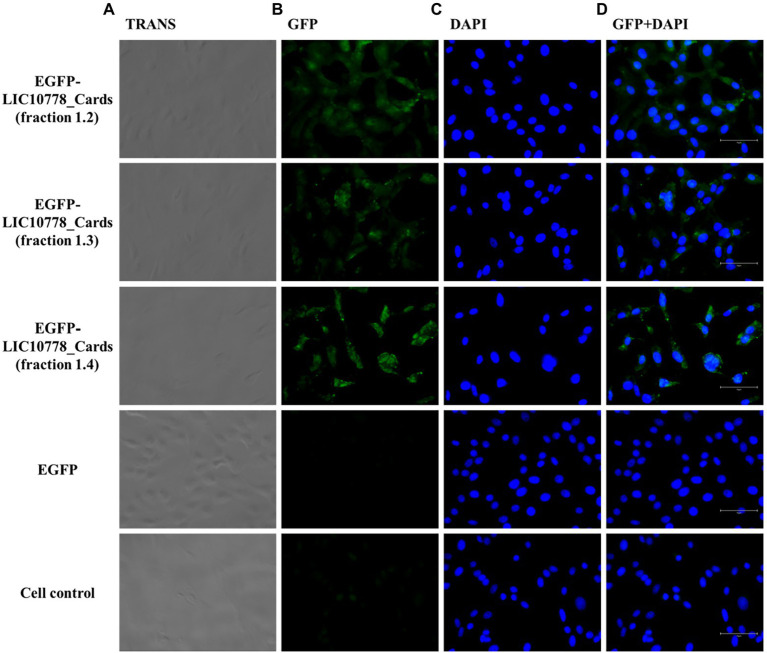
EGFP-LIC10778_Cards binding to VERO cells. Purified samples of EGFP-LIC10778_Cards protein were incubated with VERO cells, previously cultured for 5 h in serum-free media. Following 2 h incubation with the fluorescent proteins, including recombinant EGFP as negative control, cells were washed, fixed in 4% PFA, and incubated with DAPI for nuclear staining. Resulting fluorescence was observed under a fluorescence microscope (EVOS M5000, Thermo Fisher Scientific), considering available filter TRANS (transmitted light; **A)**, GFP **(B)** and DAPI **(C)**, and image magnification to 400x. In **(D)**, overlap of the same images from GFP and DAPI filters are represented, and white bars on the bottom right of each image represent 75 μm in size. Cell control represents non-treated cells. Results were obtained from two independent assays.

**Figure 8:**
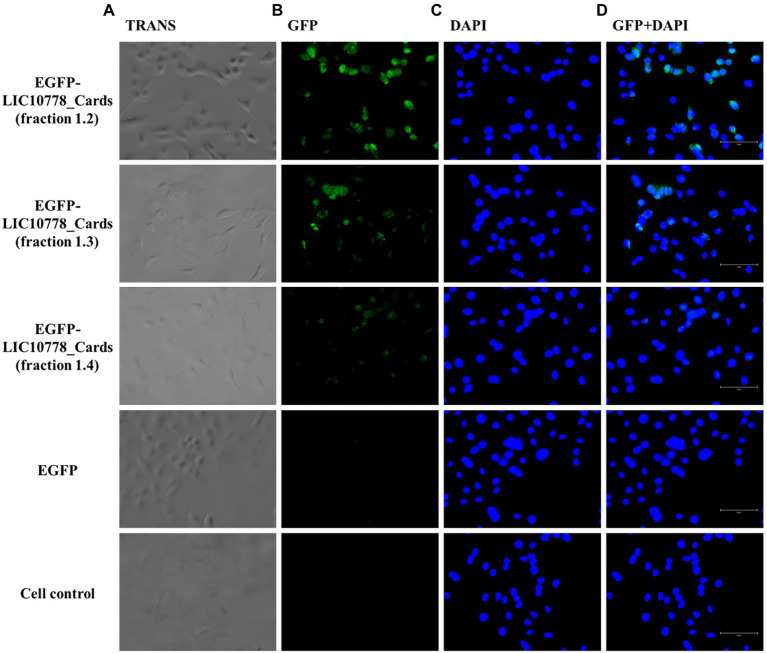
EGFP-LIC10778_Cards binding to A549 cells. Purified samples of EGFP-LIC10778 fused protein were incubated with A549 cells, previously cultured for 5 h in serum-free media. Following 2 h incubation with fluorescent proteins, including recombinant EGFP as negative control, cells were washed, fixed in 4% PFA, and incubated with DAPI for nuclear staining. Resulting fluorescence was observed under a fluorescence microscope (EVOS M5000, Thermo Fisher Scientific), considering available filter TRANS (transmitted light; **A)**, GFP **(B),** and DAPI **(C)**, and image magnification to 400×. In **(D)**, overlap of the same images from GFP and DAPI filters are represented, and white bars on the bottom right of each image represent 75 μm in size. Cell control represents non-treated cells. Results were obtained from two independent assays.

After incubating each cell with the recombinant proteins, cells were washed, fixed, and observed under a fluorescence microscope. Samples of EGFP-LIC10778_Cards showed evident interaction with both VERO and A549 cells in varying degrees (Figures 7, 8), indicating that the N-terminal region of LIC10778 protein has binding capabilities to the cell lines tested, a feature that could possibly be extended to other members of PF07598 protein family.

## Discussion

Characterization of virulent bacteria *Leptospira interrogans* regarding its interaction with different hosts remains poorly understood. The phage display technique has proved to be a valuable tool to identify new adhesins and other proteins that participate in the spirochete-host interactions (Coburn et al., 1999; Antonara et al., 2007; Rosander et al., 2011; Ching et al., 2012; Lima et al., 2013; Evangelista et al., 2014). In the present study, leptospiral phage display library was analyzed against VERO kidney epithelial cells, a continuous cell line established from the African green monkey kidney, being chosen because they represent the main organ of leptospiral colonization in the hosts (Picardeau, 2017; Samrot et al., 2021). This cell line has been extensively used in the study of several emerging pathogens, due to its high susceptibility to viruses and bacterial toxins, such as diphtheria toxin, heat-labile enterotoxins, and Shiga-like toxins (also known as “verotoxins”) (Sheets, 2000; Naoki et al., 2014). In addition, VERO cells have also been used as a model to study potential virulence factors in leptospires (Lee et al., 2002; Lima et al., 2013; Chaurasia and Sritharan, 2020; Paratsaphan et al., 2020). To recover cell-bound phages from VERO cells, we used the BRASIL single-step centrifugation approach. This methodology showed an increase in the recovery of specific phages and most importantly, a significant reduction in the background and unspecific interactions (Giordano et al., 2001). The BRASIL technique allowed the identification of relevant ligands in bacteria and other pathogens, including leptospires (Lionakis et al., 2005; Choi and Park, 2011; Lima et al., 2013; de Oliveira et al., 2016; Teixeira et al., 2021). Through this technique, the conserved hypothetical protein LIC10778 belonging to the Virulence Modifying (VM) protein family was identified and characterized in this study.

The initial evaluation of the *lic10778* gene expression by RT-qPCR revealed a significant increase in its expression in liver samples from susceptible mice after 3 days of infection by *L. interrogans* serovar Copenhageni strain Fiocruz L1-130, in agreement with the data in the literature. Lehmann and colleagues demonstrated upregulation in the expression of *la3388* gene (*lic10778* orthologous) by RT-qPCR in blood and liver samples, and to a lesser extent in kidney samples collected from hamsters in a model of acute infection by *L. interrogans* serovar Lai strain 56,601. The study also demonstrated the increase in the expression of genes of other members belonging to the PF07598 protein family, using the same samples from infected animals (Lehmann et al., 2013). Another group revealed a strong 6 to 11-fold induction in the expression of two genes encoding PF07598 proteins (*lic12339* and *lic12340*, respectively) in cultures of *L. interrogans* serovar Copenhageni strain Fiocruz L1-130 in EMJH medium under physiological osmolarity (Matsunaga et al., 2007). With regard to the *lic12339* gene, high expression levels were detected in Human Kidney-2 (HK-2) cells after 4 h of infection by *L. interrogans* serovar Copenhageni strain Fiocruz L1-130 (Chou et al., 2014) and, more recently, differential expression analyses using RNA-seq revealed the same gene as one of the most significantly expressed when pathogenic bacteria of *Leptospira borgpetersenii* serovar Hardjo strain JB197 were cultured at 37°C (Putz et al., 2021).

Prediction of three-dimensional structures and possible domains of LIC10778 protein showed a N-terminal portion being more reliably modeled from its alignment with the ricin B lectin domain. This domain is present in several proteins that interact with carbohydrates, such as AB toxins, glycosidases, and proteases from plants and bacteria. Currently, the ricin B lectin domain is annotated only in the LIC12340 protein in *L. interrogans* (Fiocruz L1-130 strain), according to the InterPro database. However, the presence of QxW repeats characteristic of the ricin B-like lectin domain (Rutenber et al., 1987; Hazes, 1996) in most of the proteins analyzed is an indicative of a similar function of this domain among all members of the PF07598 protein family (Supplementary Figure S2). Interestingly, Lehmann and colleagues found a mutation in the gene orthologous to the gene encoding LIC10778 protein after attenuation of *L. interrogans* bacteria in *in vitro* subcultures (G72E), which occurred in the possible ricin B lectin domain in the protein, suggesting the importance of this structure in the virulence of pathogenic leptospires.

Other remarkable domains present in the LIC10778 structure prediction analysis were 4tlv and 4tlw, corresponding to the CARDS toxin of *Mycoplasma pneumoniae*. CARDS toxin is a virulence factor with ADP-ribosylation and vacuolization activities in airway cells, leading to cell death. The structure of the toxin is triangular, composed of the N-terminal domain (D1) with mART active site (mono-ADP-ribosyltransferase), followed by two domains (D2 and D3) responsible for binding and internalization of the toxin in cells (Becker et al., 2015). D2 and D3 domains are also structured similarly to the ricin B lectin domain, and the similarity between these domains with the N-terminal portion of LIC10778 protein, including the partial conservation of the aromatic patch characteristic of the D3 domain of CARDS toxin, suggests that leptospiral LIC10778 may play a similar role in cell binding and internalization through similar mechanisms.

PF07598 protein family is one of the thousands of protein families that lack homologs with known structure for more accurate modeling comparison. However, computational methods used for prediction of protein structures from amino acid sequences became increasingly numerous and accessible at the end of the 20th century, facilitating and guiding functional study of proteins with functions and structures still unknown. The continuous increase in genome sequencing also makes it possible to predict accurate residue-waste contacts using evolutionary data (Anfinsen, 1973; Ovchinnikov et al., 2017). Renowned servers for protein modeling used in the present study (Phyre2, SWISS-Model, and HHpred) use structure prediction methods based on models known for homology and/or fold recognition, generating models with similar characteristics to each other, but still show regions of low structural confidence. The additional analysis of the PF07598 protein family using a recent methodology based on the formation of a three-way neural network called RoseTTAFold (Baek et al., 2021) allowed the generation of more accurate models (Supplementary Figure S3) and, together with the generated models on other servers, reinforce the evidence that proteins of this family in pathogenic leptospires may be possible virulence factors.

Prediction of three-dimensional structures and domains helped in the design of LIC10778 protein fragments for subsequent functional analysis. Using the Far Western (Overlay) Blotting technique, marked interactions were observed mainly between the LIC10778 fragments corresponding to the sequence identified by phage display (LIC10778_Phg) and the complete N-terminal region (LIC10778_D1 and LIC10778_Cards) with ECM proteins such as laminin, type I and IV collagens, plasma and cellular fibronectins and vitronectin. These results, in addition to the information available in literature, structural predictions of the N-terminal region of LIC10778 protein, and previous phage display assay followed by phage selection in VERO cells, strongly suggest that the protein may contribute to the pathogen-host interaction during leptospiral infection. Although the interaction regions between LIC10778 protein fragments and different ECM proteins in this study remain to be elucidated, it is possible to observe differences between the interactions depending on the LIC10778 region. From the results obtained, the fragment corresponding to LIC10778_Phg may represent the main carbohydrate-binding site of the entire LIC10778 protein. Therefore, this study characterizes the possible functional activities of different regions of LIC10778 protein, thus contributing to the elucidation of this unique family of PF07598 paralogous proteins in virulent leptospires.

The hypothesis that PF07598 family proteins act as toxins during the infection of pathogenic leptospires was also explored and confirmed in the very recent publications by Chaurasia et al. The group had previously described that rLA3490 protein from *L. interrogans* serovar Lai, orthologous to the LIC10695 protein in Fiocruz L1-130 strain, presented greater gene expression in samples of blood, liver, and kidneys from hamsters in an acute infection model (Lehmann et al., 2013). rLA3490 recombinant protein was shown to share the same glycoconjugate-binding specificity as the ricin B chain, and also displays cytopathic effects on HeLa cells, including cell rounding and detachment, actin depolymerization and nuclear fragmentation, leading to cell lysis and death (Chaurasia et al., 2022a). These data are the first indications of the cytotoxicity of this protein, a function that can be performed by other members of the PF07598 protein family. Furthermore, the same group showed that immunizations of susceptible animals with only the ricin B lectin domain portion of LA3490 were sufficient to significantly reduce the bacterial load in kidney and liver, despite resulting in the development of severe clinical symptoms after lethal challenge. On the other hand, immunizations with a mix of 2 or 5 full-length recombinant VM proteins (PF07598) not only reduced bacterial load in the same organs, but also resulted in the absence of clinical signs of severe leptospirosis (Chaurasia et al., 2022b), highlighting the promising potential of this protein family in the development of new vaccines against leptospirosis.

Interestingly, in a parallel investigation, we identified LIC10778 protein by shotgun phage display technique, and following a similar rationale of Chaurasia and colleagues, we have also performed its three-dimensional structure predictions, in addition to the design and expression of EGFP-LIC10778_Cards protein to evaluate the interaction of LIC10778 N-terminal domain with different mammalian cell lines, such as VERO and A549 cells, representing main spots of leptospiral infection and disease development in the hosts. We demonstrated that LIC10778 is a protein organized in AB domains, being the N-terminal fragment responsible for binding to cellular receptors, a possible feature showed by other members of the PF07598 protein family. An intriguing exception is LIC12985 protein, which is the only truncated member that lacks the N-terminal-binding domain present in all other members of PF07598 protein family in *L. interrogans* strain Fiocruz L1-130.

It is likely that LIC10778 protein and other members of PF07598 protein family, including LIC12985, have toxic effect on cells as shown for LA3490 protein (Chaurasia et al., 2022a). Therefore, the important role of this protein family in the pathology of leptospirosis is now opened to be explored in further studies.

## Data availability statement

Publicly available datasets were analyzed in this study. This data can be found at: https://www.ncbi.nlm.nih.gov/nucleotide?term=txid267671.

## Ethics statement

The animal study was reviewed and approved by Committee on Ethics in the Use of Animals - Butantan Institute (CEUAIB protocol number 1255/14).

## Author contributions

FL-F and PH conceived and designed the experiments and wrote the paper. FL-F, AT, JS, PA, and AB performed the experiments. PH, RG, AB, and MA analyzed the data and contributed to the analysis tools. All authors contributed to the article and approved the submitted version.

## Funding

This work was financially supported by Fundação de Amparo à Pesquisa do Estado de São Paulo (Fapesp 2013/15155–0 to FL-F), the Brazilian National Research Council (CNPq 164209/2015–8 to FL-F and CNPq 305430/2019–0 to PH) and Fundação Butantan (CNPq 148645/2013-5).

## Conflict of interest

The authors declare that the research was conducted in the absence of any commercial or financial relationships that could be construed as a potential conflict of interest.

## Publisher’s note

All claims expressed in this article are solely those of the authors and do not necessarily represent those of their affiliated organizations, or those of the publisher, the editors and the reviewers. Any product that may be evaluated in this article, or claim that may be made by its manufacturer, is not guaranteed or endorsed by the publisher.
