# Supplementary material for: Characterization of a virulence-modifying protein of Leptospira interrogans identified by shotgun phage display
Source: Front Microbiol. 2022 Nov 28;13:1051698. doi: 10.3389/fmicb.2022.1051698 (PMC9742253; doi:10.3389/fmicb.2022.1051698)
Supplement: Supplementary file 1 [file Data_Sheet_1.docx]

**Supplementary Material**

**Table S1. Sequences of oligonucleotide pairs used as components of qPCR reactions, cloning and sequencing assays.**

| **Target** | **Application** | **Oligonucleotide sequences** |
| --- | --- | --- |
| pG8SAET phagemid | Sequencing assays | *F*^1^: 5’-ATCTGGTGGCGTAACACCTG-3’  *R*^2^: 5’-TATTCGGTCGCTGAGGCTTG-3’ |
| *lic10778* | qPCR | *F*: 5’-GGTCTGGGCTGCCATTGAAT-3’  *R*: 5’-TCCTTCTCCACTACAATCCCT-3’ |
| *lipL32* | qPCR | *F*: 5’-GGCGATTTGGTCAGGCATAA-3’  *R*: 5’-CCGTAATCGCTGAAATGGGA-3’ |
| *16S* | qPCR | *F*: 5’-TTCAGTTGGGCACTCGTAAG-3’  *R*: 5’-CGTGTGTTGCCCTAGACATAA-3’ |
| LIC12339F | Cloning and sequencing assays | *F*: 5’-TCTTCATCAAAAGCTAATTATTCG-3’  *R*: 5’-TTACTGCAGAATACATCTACC-3’ |
| LIC10870F | Cloning and sequencing assays | *F*: 5’-GATGCTTCATCAAAAATTGAA-3’  *R*: 5’-TTAAAATGGCAGAGCACACCT-3’ |
| LIC10778F | Cloning and sequencing assays | *F*: 5’-TCATCGAATGATATTCCTAGT-3’  *R*: 5’-TTACATCAATGTGCATCTACC-3’ |
| LIC10778_LecB | Cloning and sequencing assays | *F*: 5’-CAAAGACCTACTGATAAACCA-3’  *R*: 5’-ATCTTTTAACCGATAACGCTT-3’ |
| LIC10778_Phg | Cloning and sequencing assays | *F*: 5’-TATAATTGGTATGCCTATATT-3’  *R*: 5’-ATAGAGCGATCCACTCACTGG-3’ |
| LIC10778_D1 | Cloning and sequencing assays | *F*: 5’-CAAAGACCTACTGATAAACCA-3’  *R*: 5’-AACACCCCAATTAGGTCCATA-3’ |
| LIC10778_Cards/  EGFP-LIC10778_Cards | Cloning and sequencing assays | *F*: 5’-CAAAGACCTACTGATAAACCA-3’  *R*: 5’-GTAACGGACCCAATTTAATAA-3’ |
| LIC10778_Ct | Cloning and sequencing assays | *F*: 5’-TTACTTCATACCTTCCAGATG-3’  *R*: 5’-AGAGATCATGAAATCAAAAAT-3’ |
| ^1^F: sense oligonucleotide sequences (forward).  ^2^R: antisense oligonucleotide sequences (reverse). | | |

**Table S2. Online tools used for analysis of *L. interrogans* DNA and protein sequences from shotgun phage display library and phage sequences selected by biopanning against VERO cells.**

| **Applications and tools** | **Website** | **References** |
| --- | --- | --- |
| **DNA/protein sequence alignment** |  |  |
| BLASTx/BLASTp | blast.ncbi.nlm.nih.gov/Blast.cgi | (Altschul et al., 1990; Boratyn et al., 2013) |
| Clustal Omega | www.ebi.ac.uk/Tools/msa/clustalo | (Madeira et al., 2019) |
| **Translation tool** |  |  |
| EMBOSS Transeq | www.ebi.ac.uk/Tools/st/emboss_transeq | (Madeira et al., 2019) |
| **Prediction of transmembrane regions** |  |  |
| TMHMM Server | www.cbs.dtu.dk/services/TMHMM | (Krogh et al., 2001) |
| **Prediction of signal peptides** |  |  |
| Phobius | phobius.sbc.su.se | (Käll et al., 2007) |
| SignalP | www.cbs.dtu.dk/services/SignalP-5.0 | (Petersen et al., 2011; Armenteros et al., 2019b) |
| TargetP | www.cbs.dtu.dk/services/TargetP-2.0 | (Armenteros et al., 2019a) |
| **Prediction of subcellular localization** |  |  |
| CELLO | cello.life.nctu.edu.tw | (Yu et al., 2004) |
| Gneg-mPLoc | www.csbio.sjtu.edu.cn/bioinf/Gneg-multi | (Shen and Chou, 2010) |
| LocTree3 | rostlab.org/services/loctree3 | (Goldberg et al., 2014) |
| PSORTb | www.psort.org/psortb/index.html | (Yu et al., 2010) |
| SecretomeP | www.cbs.dtu.dk/services/SecretomeP | (Bendtsen et al., 2005) |
| **Described domains** |  |  |
| InterPro | www.ebi.ac.uk/interpro | (Mitchell et al., 2019) |

**Table S3. Online tools used for prediction and analysis of LIC10778 three-dimensional structure.**

| **Tool** | **Website** | **References** |
| --- | --- | --- |
| SWISS-MODEL | swissmodel.expasy.org | (Guex et al., 2009; Bertoni et al., 2017; Bienert et al., 2017; Waterhouse et al., 2018; Studer et al., 2020) |
| Phyre2 | sbg.bio.ic.ac.uk/phyre2 | (Kelley et al., 2015) |
| Hhpred | toolkit.tuebingen.mpg.de/tools/hhpred | (Hildebrand et al., 2009; Zimmermann et al., 2018) |
| PyMol | pymol.org/2 | (Delano, 2002) |
| UCSF Chimera | cgl.ucsf.edu/chimera | (Pettersen et al., 2004) |
| Robetta (RoseTTAFold deep learning tool) | robetta.bakerlab.org | (Baek et al., 2021) |

**Table S4. Analysis of LIC10778 and other PF07598 proteins in *L. interrogans* serovar Copenhageni strain Fiocruz L1-130 (except LIC12985). The presence of signal peptide, transmembrane region and subcellular location were predicted using the online tools Phobius, TMHMM and LocTree3, respectively.**

| **Protein** | **Presence of signal peptide** | **Presence of transmembrane region** | **Subcellular localization** |
| --- | --- | --- | --- |
| **LIC10778** | **Yes** | **Yes** | **Secreted (97%)** |
| LIC12791 | Yes | Yes | Secreted (97%) |
| LIC12986 | Yes | Yes | Secreted (98%) |
| LIC12715 | Yes | Yes | Secreted (97%) |
| LIC10695 | Yes | No | Secreted (98%) |
| LIC10870 | Yes | Yes | Secreted (98%) |
| LIC10639 | Yes | No | Secreted (98%) |
| LIC12844 | Yes | Yes | Secreted (98%) |
| LIC12963 | Yes | Yes | Secreted (98%) |
| LIC11358 | Yes | No | Secreted (97%) |
| LIC12340 | Yes | No | Secreted (98%) |
| LIC12339 | No | Yes | Secreted (98%) |

**Figure S1. Multiple sequence alignment of PF07598 protein family.** Overview of the multiple alignment of 13 proteins clustered by the DUF1561 found in *L. interrogans* serovar Copenhageni strain Fiocruz L1-130, with color code based on percentage of identity between sequences. Colored boxes delimit the following features: signal peptides (green), sequence identified by phage display in LIC10778 (yellow), and 12 conserved cysteine ​​residues throughout the protein sequences (red). Multiple alignment obtained by Clustal Omega tool and analyzed by UGene software.

**Figure S2. N-terminal analysis of LIC10778 and other PF07598 proteins. (A)** Multiple alignment of the N-terminal domains of the PF07598 protein family found in the *L. interrogans* serovar Copenhageni strain Fiocruz L1-130, considering the region corresponding to the lectin B domain, and with color code based on the percentage of identity among sequences. Interestingly, this ricin B lectin domain is only annotated in the LIC12340 protein, according to the InterPro database (green arrow; residues 40 to 157 of LIC10778 protein). Boxes delimit the QxW or similar repeats characteristic of lectin B domain (in red) and four conserved cysteine residues in all protein sequences (in yellow). Multiple alignment obtained by Clustal Omega tool and analyzed by UGene software. **(B)** Representation of the lectin B domain of abrin (PBD 1abr_B; *left*, in golden yellow), model of the N-terminal region of LIC10778 protein generated by Phyre2 server and corresponding to the lectin B domain (*right*, in cyan blue) and alignment of the two domains (center). Modified representations in PyMol software to highlight QxW repeats (in red) and cysteine residues/disulfide bridges (in white). Calculation of RMSD in angstroms performed by UCSF Chimera software.

**Figure S3. Three-dimensional models of the PF07598 protein family in Fiocruz L1-130 strain, generated by the Robetta server (RoseTTAFold methodology).** Modified representations in PyMol software for delimitation of possible protein domains: signal peptide (in red), alignment with D2 domain of CARDS toxin (in cyan blue), sequence identified by phage display (in pink), alignment with D3 domain of CARDS toxin (in yellow) and C-terminal portion of the proteins (in green). Models with errors smaller than 5 angstroms and confidence index ranging from 0.65 to 0.76.
