# Supplementary figures and images for: Characterization of a virulence-modifying protein of Leptospira interrogans identified by shotgun phage display
Source: Front Microbiol. 2022 Nov 28;13:1051698. doi: 10.3389/fmicb.2022.1051698 (PMC9742253; doi:10.3389/fmicb.2022.1051698)

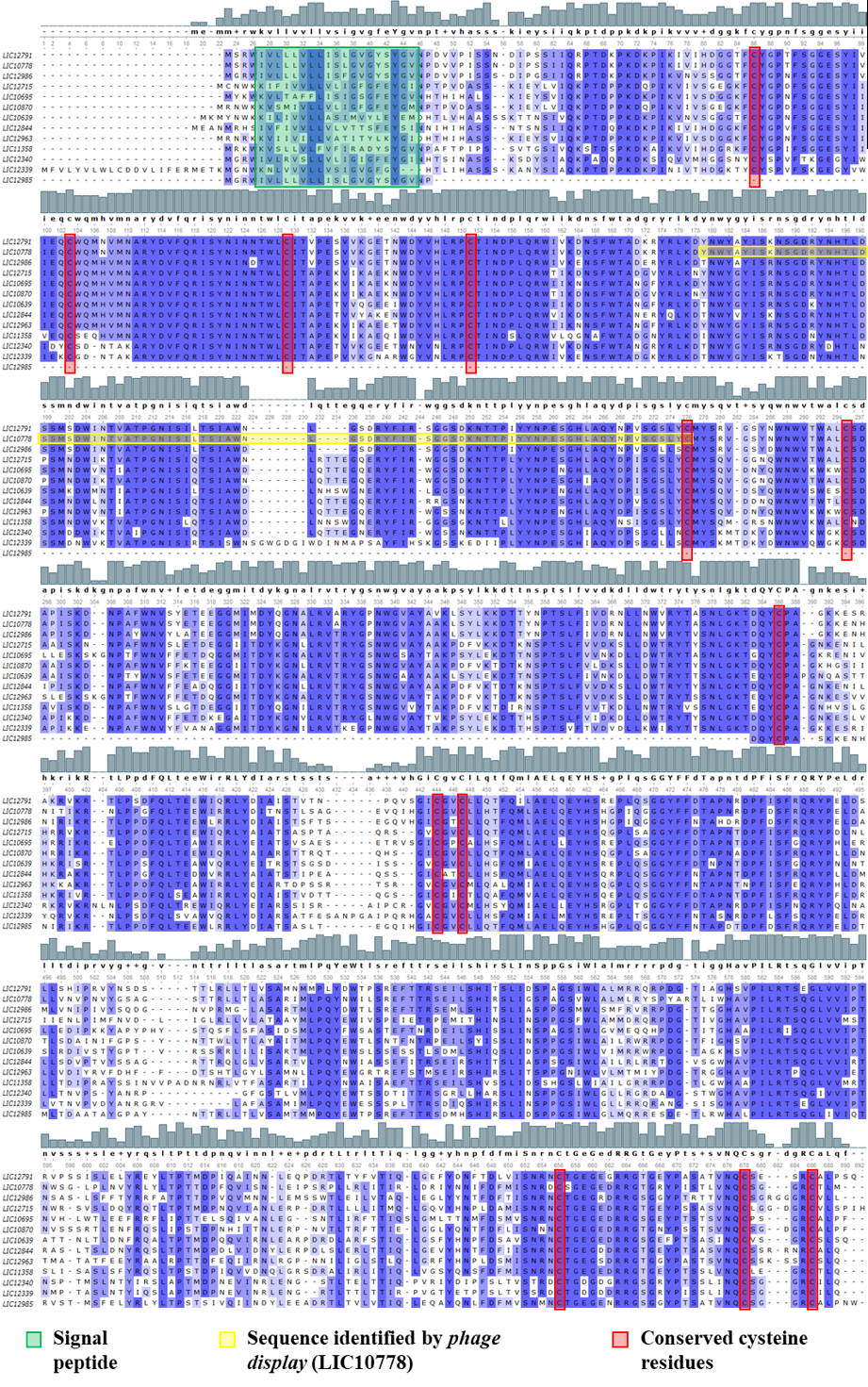

Supplement: Supplementary file 2 [file Image_1.TIF]

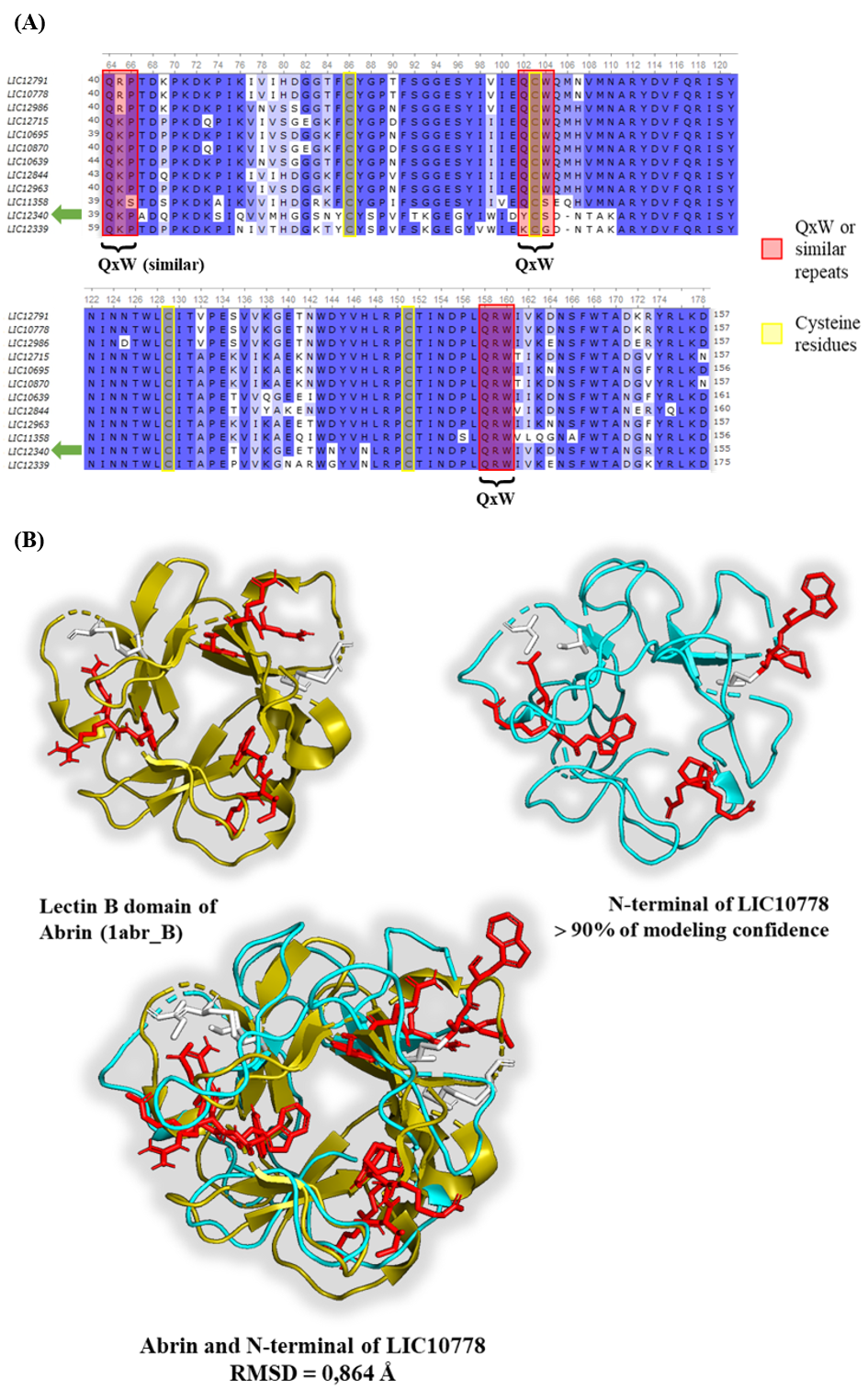

Supplement: Supplementary file 3 [file Image_2.TIF]

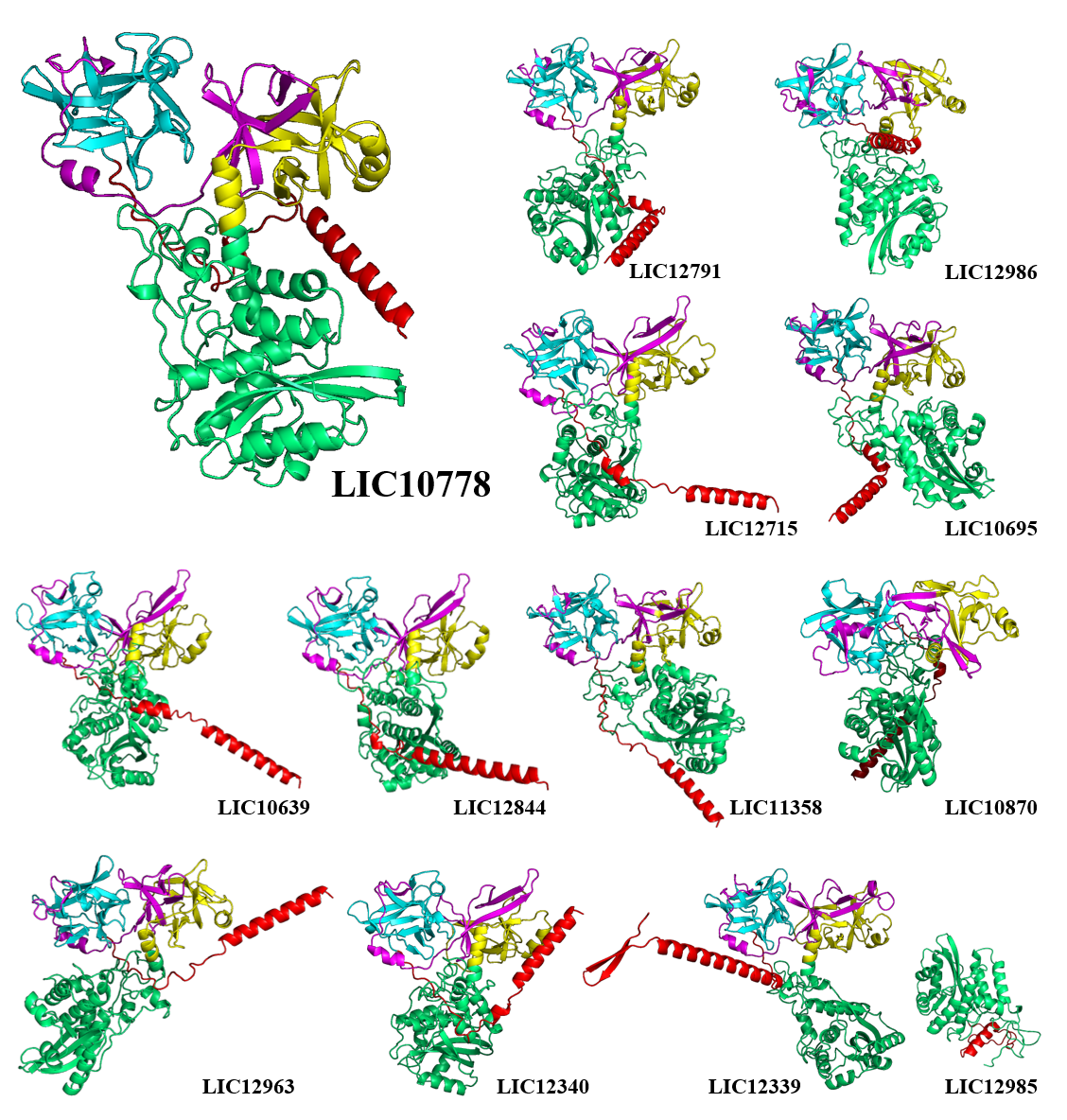

Supplement: Supplementary file 4 [file Image_3.TIF]
